# Gene expression profiles uncover individual identities of gnathal neuroblasts and serial homologies in the embryonic CNS of *Drosophila*

**DOI:** 10.1242/dev.133546

**Published:** 2016-04-15

**Authors:** Rolf Urbach, David Jussen, Gerhard M. Technau

**Affiliations:** Institute of Genetics, University of Mainz, Mainz D-55099, Germany

**Keywords:** Central nervous system, Neuroblasts, Segmental patterning, *Drosophila* brain, Gene expression profile, *Deformed*

## Abstract

The numbers and types of progeny cells generated by neural stem cells in the developing CNS are adapted to its region-specific functional requirements. In *Drosophila*, segmental units of the CNS develop from well-defined patterns of neuroblasts. Here we constructed comprehensive neuroblast maps for the three gnathal head segments. Based on the spatiotemporal pattern of neuroblast formation and the expression profiles of 46 marker genes (41 transcription factors), each neuroblast can be uniquely identified. Compared with the thoracic ground state, neuroblast numbers are progressively reduced in labial, maxillary and mandibular segments due to smaller sizes of neuroectodermal anlagen and, partially, to suppression of neuroblast formation and induction of programmed cell death by the Hox gene *Deformed*. Neuroblast patterns are further influenced by segmental modifications in dorsoventral and proneural gene expression. With the previously published neuroblast maps and those presented here for the gnathal region, all neuroectodermal neuroblasts building the CNS of the fly (ventral nerve cord and brain, except optic lobes) are now individually identified (in total 2×567 neuroblasts). This allows, for the first time, a comparison of the characteristics of segmental populations of stem cells and to screen for serially homologous neuroblasts throughout the CNS. We show that approximately half of the deutocerebral and all of the tritocerebral (posterior brain) and gnathal neuroblasts, but none of the protocerebral (anterior brain) neuroblasts, display serial homology to neuroblasts in thoracic/abdominal neuromeres. Modifications in the molecular signature of serially homologous neuroblasts are likely to determine the segment-specific characteristics of their lineages.

## Abstract (precis)

**Highlighted article:** Characterisation of the neural stem cells in the gnathal head region completes the mapping of all individual neuroblasts that generate the *Drosophila* CNS.

## INTRODUCTION

The development of the central nervous system (CNS) in *Drosophila* begins with the formation of a stereotyped population of neural stem cells, termed neuroblasts (NBs), which delaminate from the neuroectoderm in a precise spatiotemporal pattern. Positional cues within the neuroectoderm provided by products of early regulatory genes control the identity of embryonic NBs (e.g. reviewed by Skeath and Thor, 2003). Each NB within a hemisegment acquires a unique identity that is reflected in the typical developmental time point and position of its delamination from the neuroectoderm, the combinatorial code of genes it expresses (Hartenstein and Campos-Ortega, 1984; Doe, 1992; Urbach and Technau, 2003b), and the production of a specific cell lineage (Bossing et al., 1996; Schmidt et al., 1997; Schmid et al., 1999). The developmental patterns and identities of embryonic NBs have been described in detailed maps for the brain (Younossi-Hartenstein et al., 1996; Urbach et al., 2003; Urbach and Technau, 2003a,b) and the thoracic (T1-T3) and anterior abdominal neuromeres (A1-A7) of the ventral nerve cord (VNC) (Doe, 1992; Broadus et al., 1995). In contrast to the brain, neuromeres T1-A7 originate from a rather stereotypic array of ∼30 NBs per hemisegment. In each hemisegment, the Cartesian grid-like expression of anteroposterior (AP) and dorsoventral (DV) patterning genes is virtually identical. Hence, NBs developing from the same ‘quadrants’ in different segments represent serial homologs, as they acquire a similar identity (reviewed by Skeath and Thor, 2003; Technau et al., 2006). Recently, NB patterns were also established for the derived terminal abdominal neuromeres (A8-A10), and were shown to consist of segment-specifically reduced sets of serial homologous NBs (Birkholz et al., 2013a). Accordingly, the gnathal neuromeres, which constitute the subesophageal zone (between brain and truncal neuromeres) comprise the last region of the fly CNS in which patterns and identities of NBs have not been resolved so far. The labial (LB), maxillary (MX) and mandibular (MN) neuromeres (from posterior to anterior) develop in the gnathal part of the head, in which segmental units are more evident compared with the pregnathal head from which the embryonic brain arises (Schmidt-Ott and Technau, 1992; Urbach and Technau, 2003a). During ongoing development, these neuromeres fuse to become the subesophageal zone at the posteriormost site of the adult brain (Ito et al., 2014), a region implicated in feeding and the taste response (e.g. Scott et al., 2001; Gendre et al., 2004; Melcher and Pankratz, 2005).

In this study, we have undertaken a comprehensive survey of the early development of the gnathal neuromeres. We traced the spatiotemporal pattern of formation of the gnathal NBs. Within the final NB pattern by stage 11, numbers are progressively diminished in LB, MX and MN (i.e. in posterior-anterior order), as compared with thoracic neuromeres. This is mainly due to smaller segmental sizes of gnathal neuroectodermal anlagen, modifications in patterning gene expression, and by the activity of Deformed (Dfd), which supresses NB formation. Moreover, we provide comprehensive maps of the ∼76 gnathal NBs (within the three hemisegments, plus three unpaired midline NBs), which reveal the expression patterns of 46 different marker genes (encoding 41 transcription factors), that are specifically expressed in particular NB subsets and form combinatorial codes specifying each NB individually. Thus, detailed spatiotemporal and molecular maps now exist for the entire population of NBs giving rise to the brain (except for the optic lobes) and VNC of the fly (in total 2×567 NBs of neuroectodermal origin). The completed map makes it possible, for the first time, to compare the patterns and molecular characteristics of segmental populations of NBs throughout the CNS and to identify serial homologies. Our data demonstrate that almost all gnathal NBs are serially homologous to NBs in more posterior segments, and provide support that most NBs in the tritocerebrum, and about half of the NBs in the deutocerebrum, show serial homology to NBs in the VNC.

This study provides a basis for investigating, at the level of identified NBs and their lineages, the mechanisms that underlie the structural and functional diversification of the segmental CNS units. It will also facilitate comparisons of the patterns and molecular profiles of neural stem cells among different species in the context of evolutionary investigations.

## RESULTS

### Spatiotemporal pattern of NB formation in the gnathal head segments

**Fig. 1.**
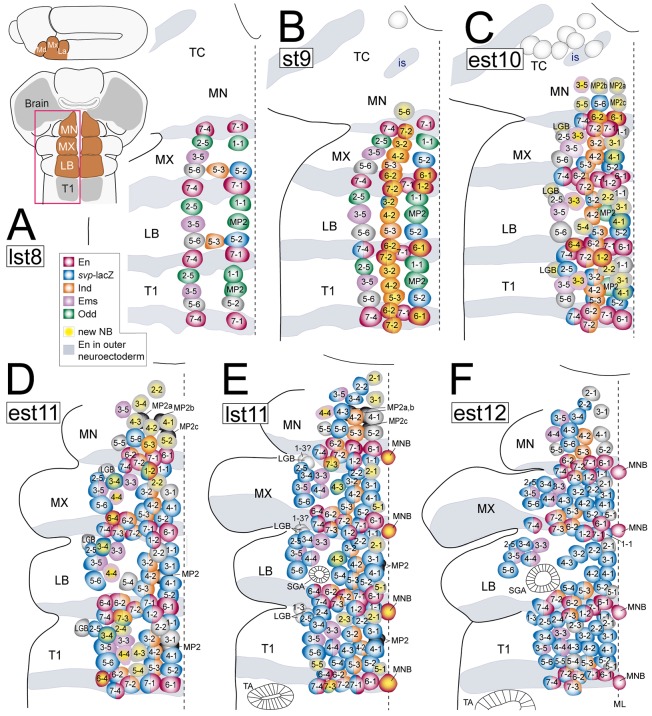
**Spatiotemporal development of the NB pattern in the gnathal segments.** (A-F) Semi-schematic representations of ventral views of the left half of the gnathal segments (MN, mandibular; MX, maxillary; LB, labial) and the prothoracic segment (T1), as framed in the inset in A. Typical NB arrangement is shown at (A) late stage 8 (lst8), (B) stage 9 (st9), (C) early stage 10 (est10), (D) early stage 11 (est11), (E) late stage 11 (lst11) and (F) early stage 12 (est12). The position, formation time point and expression of five marker genes (see color code) allows the identification of individual NBs. NB formation is significantly delayed in MN, similar to observations made in tritocerebrum (TC; NBs light gray) (Urbach et al., 2003). Existence of NB1-3 is unclear in MX and LB (see E and Fig. S2.5). (F) Dorsal labial NBs become separated by salivary gland anlagen (SGA). is, intercalary *en* stripe; MNB, median neuroblast; ML, ventral midline; LGB, longitudinal glioblast; TA, tracheal anlagen (see also following figures).

We traced the pattern of NBs in the gnathal segments in comparison to T1 in flat preparations of fixed *Drosophila* embryos during stages 8-12. This developmental period was subdivided into six stages, most of which match those previously reported in the truncal CNS (Hartenstein et al., 1987; Doe, 1992; Broadus et al., 1995; Birkholz et al., 2013a) and brain (Urbach et al., 2003). NBs were identified by size, subectodermal position and expression of the stem cell markers *deadpan* (*dpn*) and *worniu* (*wor*). The final NB pattern is established by late stage 11. At that stage, *dpn* and *wor* are expressed not only in NBs but also in gnathal sensory organ precursors (SOPs), some of which lie in close vicinity to dorsal NBs. The SOP-specific marker *cousin of atonal* (*cato*) (Goulding et al., 2000) allowed us to unambiguously discriminate NBs from SOPs (Fig. S1). By means of the stereotypic position and time at which each NB develops within the neuroectoderm, and expression of the five marker genes *engrailed* (*en*; indicating posterior NBs and segmental boundaries) (DiNardo et al., 1985; Doe, 1992), *intermediate neuroblasts defective* (*ind*; indicating intermediate NBs) (Weiss et al., 1998), *odd skipped* (*odd*), *seven up* (*svp*)*-lacZ* (Broadus et al., 1995) and *empty spiracles* (*ems*) (all expressed in specific NB subsets) we were able to identify individual gnathal NBs in the developing NB pattern (Fig. 1). The spatiotemporal pattern of gnathal NB development is largely invariant (among specimen). Whereas the formation of NBs in LB and MX resembles that of truncal segments, it is significantly delayed in MN, particularly in anterior positions. As compared with T1 (32 NBs per hemisegment), the final number of NBs is reduced in all gnathal segments, decreasing from LB to MN: aside the unpaired median NB (MNB), we found ∼28 NBs in LB, ∼26 NBs in MX, and 22 NBs in MN per hemisegment (Fig. 1F).

### Each gnathal NB expresses a specific combinatorial code of marker genes

**Fig. 2.**
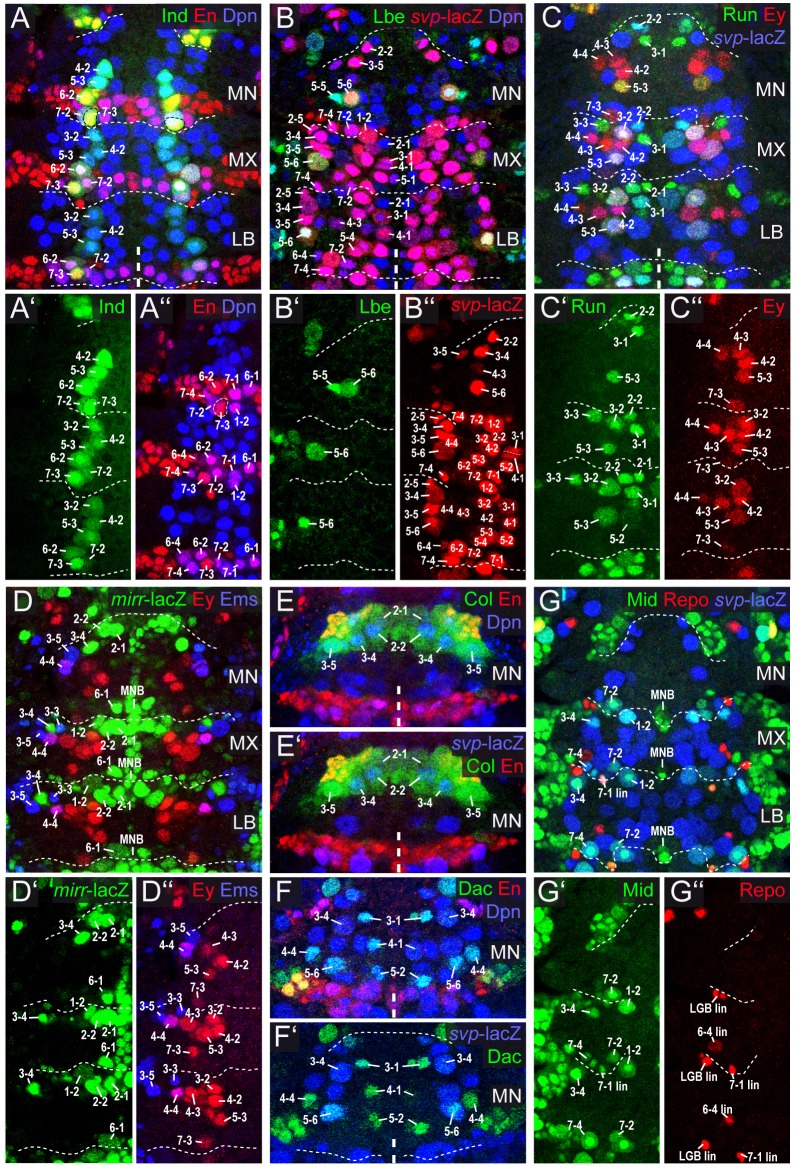
**Mapping and identification of NBs in gnathal neuromeres.** (A-G) Composite confocal images of flat preparations (ventral view) of late stage 11 embryos stained for different combinations of molecular markers as indicated. Subsets of NBs identified by marker staining(s) and position are labeled. (A′,A″,B′,B″,C′,C″,D′,D″,G′,G″) Left side. (A-A″) Ind^+^ NB3-2 is lacking in MN. (B-B″) Lbe is atypically expressed in mandibular NB5-5. (C-C″) Run is found in increasingly smaller NB subsets from LB to MN. Ey is expressed in a reduced NB subset in MN. (D-D″) *mirr*-*lacZ* is detected in reduced NB subsets in gnathal segments, and Ems in MN. (E,E′) Col is exclusively expressed in the four anteriormost mandibular NBs/hemisegment. (F,F′) Dac is exclusively expressed in MN, in four to five anterior NBs/hemisegment. (G-G″) Mid is expressed in a reduced NB subset in MN. Repo^+^ glial cells derive from NB6-4, 7-4 and LGB.

**Fig. 3.**
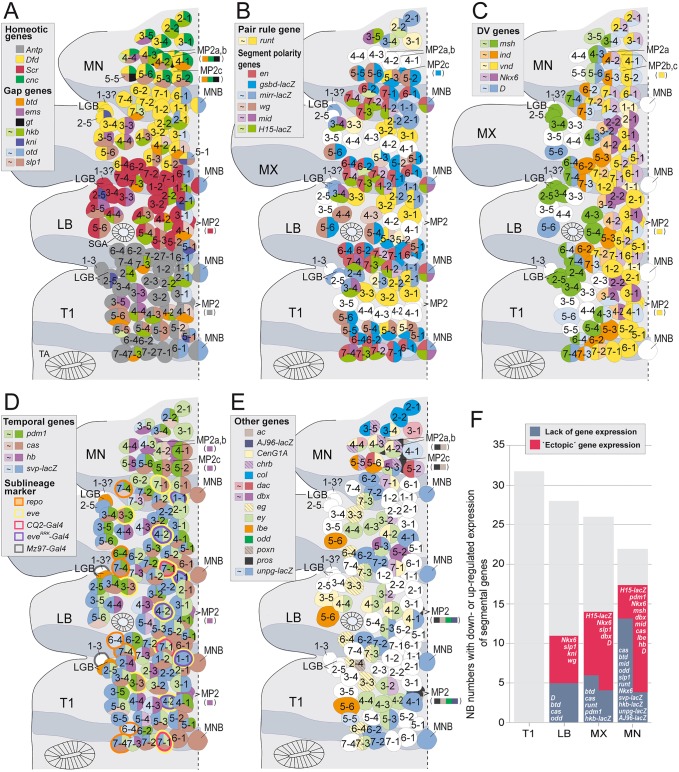
**NB maps summarizing the expression of AP and DV patterning genes, temporal genes and other NB marker genes in gnathal neuromeres compared with the prothoracic neuromere.** (A-E) Expression of subgroups of marker genes (as indicated by color code) in the full complement of gnathal NBs (left side) at late stage 11; sublineage markers for specific NBs in D are indicated by outer circles. (F) Gray columns represent the total number of NBs in the respective segments. Colored columns indicate numbers of NBs in which expression of the indicated genes is ‘ectopically′ detected (red) or lacking (blue), compared with T1. For example, in MN 14 NBs exhibit ‘ectopic’ expression of one or more of those genes (out of the ten indicated), whereas 13 NBs lack expression of one or more other genes (out of the 11 indicated). In some NBs, expression of certain genes is ‘ectopically’ detected and that of others is lacking (indicated by overlapping columns).

In order to further characterize individual gnathal NBs in our map we investigated the expression of 64 NB marker genes (using antibodies, *in situ* probes or *lacZ* lines; see Table S1), most of which have previously been used to map NBs in the thorax, abdomen and brain (Doe, 1992; Broadus et al., 1995; Younossi-Hartenstein et al., 1996; Urbach and Technau, 2003b; Birkholz et al., 2013a). Eighteen of these marker genes, expressed in subsets of brain NBs (Urbach and Technau, 2003b; Urbach, 2007; Kunz et al., 2012; data not shown), were not expressed in gnathal (or thoracic) NBs (Table S1). Thus, we identified 46 marker genes as expressed in all [*asense* (*ase*), *wor*, *dpn*] or in specific subsets of gnathal NBs (including two marker genes for typical lineage components) as shown in detail in Fig. 2 (see also Figs S2.1-S2.6) and summarized in Fig. 3. Among these we provide novel markers for NB subsets: *knirps* (*kni*), *buttonhead* (*btd*), *Dbx*, *Centaurin gamma 1A* (*CenG1A*), *charybde* (*chrb*), *collier* (*col*; *knot* – FlyBase) and *giant* (*gt*). All of the ∼76 NBs of the three gnathal hemisegments (plus three unpaired MNBs) are individually identifiable by their characteristic developmental time point, neuroectodermal origin (along AP and DV axes) (Fig. 1, Table S2) and by their unique expression profiles (Figs 2 and 3, Figs S2.1-S2.6).

### Serial homology of gnathal and thoracic/abdominal NBs

As 32 of the 46 marker genes (indicated with an asterisk in Table S1) are expressed in segmentally repeated subsets of thoracic and abdominal NBs, we used the expression of these ‘segmentally conserved’ marker genes as indicators for serial homology of gnathal NBs, in addition to the position and time point of their formation (Fig. 1, Table S2). Compared with T1 (resembling the ground state) we found these characteristics to be strongly conserved in corresponding NBs in LB and MX and, despite more pronounced modifications, largely in the MN. Only in a few cases was the serial homology of mandibular NBs ambiguous. For example, NB5-5 (lacking in MX and LB) expresses the typical markers *gooseberry* (*gsb*)*-lacZ*, *wingless* (*wg*) and *sloppy paired 1* (*slp1*), but lacks expression of *unplugged* (*unpg*)*-lacZ*, *huckebein* (*hkb*)*-lacZ* and *svp-lacZ*, and atypically expresses *chrb*, Gt, Dachshund (Dac) and Ladybird early (Lbe) (Figs 2 and 3, Figs S2.1, S2.2 and S2.4). Further, we identified ∼3 NBs per mandibular hemisegment that express an MP2-like marker gene profile of Prospero (Pros), Achaete (Ac), Hunchback (Hb) and Ventral nervous system defective (Vnd), but lack the MP2-specific markers Odd and *Aj96-lacZ* (*lacZ*^AJ96^) (Fig. 3C-E, Fig. S2.6A-C). Pros is nuclear from their time of formation and is not localized cortically during the largely symmetric division (Fig. S2.6D,E), as is characteristic for MP2 (Spana and Doe, 1995). Considering their molecular profile and division behavior and the fact that they all emerge from the same *ac*/*scute* (*sc*)-coexpressing proneural domain (see below), they might represent ‘duplicated’ serial homologs of the MP2s found in truncal segments.

Lack of marker gene expression indicated the segment-specific absence of particular gnathal NBs (compared with T1), as shown for some examples in the following (Fig. 3, Fig. S2 and as detailed in Table S2). Lack of the expression profile Dpn^+^/Pox neuro (Poxn)^+^/Eagle (Eg)^+^/*hkb*-*lacZ*^+^ revealed the absence of NB2-4, and lack of the Dpn^+^/*mirror* (*mirr*)*-lacZ*^+^/En^–^/Runt (Run)^+^ profile revealed the absence of NB2-3, both in all gnathal segments. Lack of the Dpn^+^/Eyeless (Ey)^+^/Ind^+^/Dbx^+^/Run^+^ profile demonstrated loss of NB3-2, and lack of Dpn^+^/Eg^+^/Ems^+^/*svp-lacZ*^–^/Run^+^ loss of NB3-3, both specifically in MN. Our data show that segmental modifications in the NB pattern are due to dorsal NBs missing preferentially in the anterior compartment of the gnathal segments. Accordingly, NBs 2-3, 2-4 and 5-5 are lacking in LB, and NBs 2-3, 2-4, 2-5, 5-4, 5-5 and MP2 are lacking in MX. NBs 1-3, 2-3, 2-4, 2-5, 3-2, 3-3, 5-4 and the longitudinal glioblast (LGB) are missing in MN, but so are the ventral NBs 1-1, 1-2, 5-1 and, exceptionally, the posterior NB6-4. There seems to be no correlation between NBs lacking in gnathal segments and their time point of formation in T1.

In summary, although MD shows the most significant reduction in NB numbers we identified a potential NB5-5 (missing in MX and LB) and ∼3 MP2-like NBs instead of one in the other hemineuromeres (except MX, where MP2 is missing). Based on their individual expression profiles, all NBs in LB and MX and almost all NBs in MN have serially homologous counterparts in the thoracic/abdominal segments.

### Gene expression profiles in serially homologous NBs are progressively modified from LB to MN

Next, we estimated the extent to which gene expression is modified in individual gnathal NBs by considering those 32 ‘conserved’ marker genes (indicated with an asterisk in Table S1) that are expressed in segmentally repeated subsets of thoracic/abdominal NBs (Table S2). We often observed that, compared with T1, expression of genes is lacking or they are ‘ectopically’ expressed: in total, the expression of eight genes was altered in a subset of 11 (out of 29) NBs in LB, the expression of ten genes in a subset of 14 (out of 26) NBs in MX, and the expression of 18 genes in a subset of 17 (out of 22) NBs in MN (Fig. 3F). This indicates that profiles of segmentally expressed genes in serially homologous NBs progressively differ from LB to MN. We identified further genes to be exclusively (*col*, *gt*, *chrb*) or preferentially (*dac*, *CenG1A*) expressed in particular subsets of mandibular NBs (Fig. 2E,F, Fig. 3E, Figs S2.1 and S2.4). Considering these factors altogether, the expression profile of almost all mandibular NBs is altered compared with corresponding NBs in other VNC neuromeres. Thus, the identity of many gnathal, and in particular mandibular, NBs has undergone segmental modifications.

### Identification of serially homologous NBs in the neuromeres of trunk and brain

**Fig. 4.**
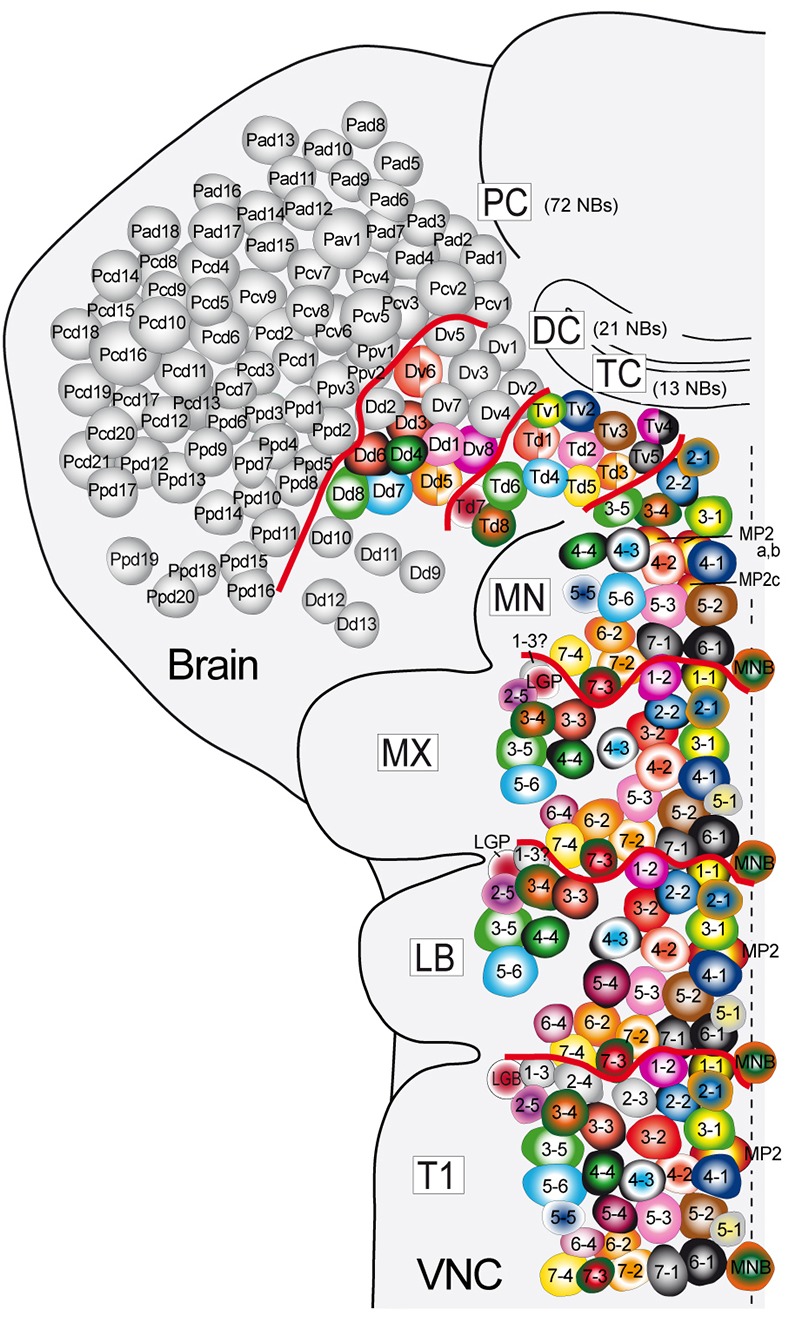
**Serial homologous NBs in neuromeres of the trunk and brain.** Assignment of potentially serially homologous NBs (same color) in neuromeres of brain (TC, tritocerebrum; DC, deutocerebrum; PC, protocerebrum), gnathal segments (MN, mandibular; MX, maxillary; LB, labial) and prothorax (T1; resembling the ground state), based on developmental time point, neuroectodermal origin (in AP and DV axes) and the specific molecular signature of individual NBs (as summarized in Table S2). A few NBs in TC and DC show serial homology to two NBs in neuromeres of ventral nerve cord (VNC). The brain NB map is according to Urbach et al. (2003).

We further investigated the extent to which individual NBs in the brain and trunk share specific developmental and molecular traits, which may support serial homology (Urbach and Technau, 2004). Although the expression of many marker genes has already been described in brain NBs (Urbach and Technau, 2003b), further markers were analyzed in brain NBs that are segmentally expressed in truncal NBs [*Nkx6* (*HGTX* – FlyBase), Dbx, *H15-lacZ*, Midline (Mid), *btd*, Ind] (Table S2; and data not shown) to allow a comprehensive comparison of their molecular signatures. When comparing the developmental time point, neuroectodermal origin and specific molecular signature of individual thoracic and gnathal NBs with those in the two posterior brain neuromeres, i.e. the tritocerebrum and deutocerebrum, remarkable parallels were observed (summarized in Fig. 4 and Table S2). Accordingly, we could attribute ten out of 13 NBs in the tritocerebrum to corresponding NBs in the thoracic and, usually, gnathal neuromeres. Each of the remaining three NBs (Td3, Tv4,5) shares features with two or three thoracic NBs, but could not be unambiguously assigned (see Table S2). Although all NBs in the tritocerebrum develop from neuroectodermal positions similar to those of their truncal homologs, most of them develop significantly later, comparable to the corresponding NBs in MN.

Applying these criteria to individual NBs in the deutocerebrum, ∼9 out of 21 NBs exhibit strong parallels with specific NBs in the thoracic and gnathal neuromeres. Usually, the gene expression profiles of deutocerebral NBs show greater similarity to those of corresponding NBs in truncal neuromeres than to those in the tritocerebrum. In some cases, strong parallels were obvious between an NB in the deutocerebrum (Dd3,4,6) and in the truncal neuromeres, but a corresponding NB was missing in the tritocerebrum. Conversely, for some NBs in the tritocerebrum (Td5,7, Tv3) that share similarities with specific NBs in the trunk, corresponding NBs could not be identified in the deutocerebrum (Fig. 4).

These data demonstrate close correspondence between individual NBs in the posterior brain (trito- and deutocerebrum) and trunk, supporting the assumption that these NBs are serially homologous. NBs in MX represent a subset of those in LB, and those in MN largely represent a subset of those in MX. Interestingly, NBs in the tritocerebrum again seem to represent a subset of NBs in MN. Although our data support the existence of serially homologous NBs also in the deutocerebrum, less than half of the deutocerebral NBs and none of the ∼70 protocerebral NBs appear to have counterparts in more posterior segments. Thus, neuromeres following T1 anteriorly exhibit an increasing degree of derivation from the ground state.

### Gnathal NB4-2 lineages reveal segment-specific modifications with regard to RP2 development

Segmental modifications in gene expression profiles of gnathal NBs are reflected by their cell lineages, as judged from the expression of sublineage markers [e.g. Even skipped (Eve), *eve^RRK^-*Gal4, *CQ-*Gal4] (Fig. 3D, Fig. S2.5). Eve and *eve^RRK^*-Gal4 are continuously coexpressed in the NB4-2-derived RP2 motoneuron in thoracic/abdominal segments (Bossing et al., 1996). In MN, however, we did not detect Eve/*eve^RRK^*>GFP^+^ cells at any stage, indicating that mandibular NB4-2 does not form RP2. By contrast, in LB and MX, we observed that RP2 is often lacking at stage 15, although consistently present at earlier stages (Fig. S2.5). In cell death-deficient *Df(3L)H99* embryos (White et al., 1994), Eve^+^ RP2 is restored to 100% in MX and LB at stage 15 (*n*=22 hemisegments), indicating that RP2 normally undergoes programmed cell death (PCD) in both segments (Fig. S2.5, Table S3). Thus, the gnathal NB4-2 lineages reveal segment-specific diversification of RP2, which does not develop in MN and undergoes PCD in MX and LB.

### PCD does not significantly account for the reduction in NB numbers in gnathal segments

**Fig. 5.**
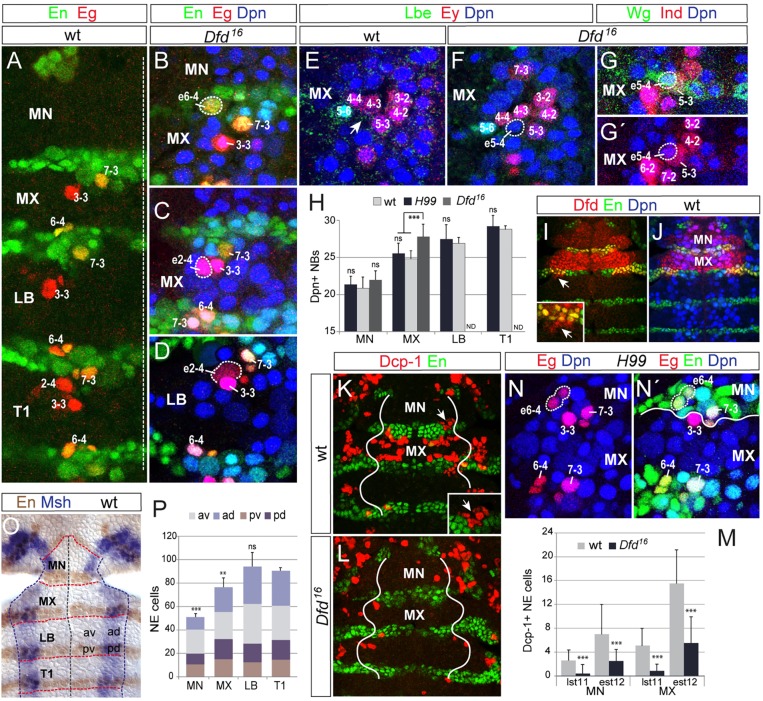
**Roles of Dfd, programmed cell death and the number of neuroectodermal progenitors in regulating NB numbers in gnathal segments.** (A-D) Gnathal (MN, MX, LB) and prothoracic (T1) left hemisegments in wild type (wt) (A) or *Dfd^16^* mutants (B-D). White dotted lines outline ectopic Eg^+^ NBs. (E,F) Lbe^+^ and Ey^+^ NBs are indicated. Note the ectopic Dpn^+^ NB5-4-like cell (e5-4) in F. (G,G′) Ectopic NB5-4 expresses row 5-specific Wg (G) and is positioned dorsally to Ind^+^ NB5-3 (G′). (H) Number of Dpn^+^ NBs in gnathal and prothoracic hemisegments in wild type (MN, 20.8±1.5; MX, 24.6±1.0; LB, 26.9±0.7; T1, 28.8±0.5; *n*=14 each), *Df(3L)H99* (*H99*; MN, 21.2±1.1; MX, 25.7±1.4; LB, 27.5±2.0; T1, 29.2±1.4; *n*=17 each) and *Dfd^16^* (MN, 21.9±0.9; MX, 27.8±1.8; *n*=16 each). ND, not determined (Dfd expression largely restricted to MN and MX). (I,J) Dfd expression in outer neuroectoderm (I) and within the NB layer (J). (I) Note the low level of Dfd in the neuroectoderm of MN compared with MX. Inset shows Dfd in anterodorsal labial neuroectodermal cells (arrow). (K-N′) At early stage 12 Dcp-1 signal is reduced in *Dfd^16^* mutant mandibular and maxillary neuroectoderm. Solid white lines indicate the dorsal border of neuromeres. (M) The average number of Dcp-1^+^ neuroectodermal (NE) cells is diminished in *Dfd^16^* MN and MX. wt, late stage 11 (lst11): MN, 2.6±1.8; MX, 5.1±3.1 (*n*=14 each). *Dfd^16^* lst11: MN, 0.4±1.6; MX, 0.8±1.2 (*n*=28 each). wt, early stage 12 (est12): MN, 7.0±5.1; MX, 15.5±5.7 (*n*=29 each). *Dfd^16^* est12: MN, 2.5±2.0; MX, 5.5±4.5 (*n*=32 each). (N,N′) Note the ectopic mandibular NB6-4 (e6-4) in *Df(3L)H99*. (O) Stage 9. Red lines indicate segmental borders, blue lines dorsal neuroectodermal border, black line the ventral midline. In LB, four spatial quadrants are indicated: ad, anterodorsal; av, anteroventral; pd, posterodorsal; pv, posteroventral. (P) Neuroectodermal cell numbers in gnathal and prothoracic hemisegments and in each quadrant of each hemisegment (color coded). *n*=7-11 (all quadrants). (A-G′,I-L,N,N′) Composite confocal images. (H,M,P) Data are mean±s.d. **P<0.01; ***P<0.0001; ns, not significant; two-tailed *t*-test.

To investigate whether PCD regulates segment-specific differences in the gnathal NB pattern we performed antibody staining against Death caspase-1 (Dcp-1), an early hallmark of cell death. During the period of NB formation, until early stage 12, Dcp-1 signal was largely absent from the labial neuroectoderm, and detected particularly in neuroectoderm of the posterior mandibular and anterior maxillary compartment; it was not detectable in gnathal NBs (Fig. 5K). This suggests that PCD does not account for the reduced NB numbers in LB.

To investigate whether NB formation is affected by PCD occurring in the posterior MN and anterior MX, we analyzed the NB patterns in *Df(3L)H99* embryos at late stage 11. The spatial arrangement and total number of Dpn^+^ NBs in all mutant gnathal segments did not obviously differ from wild type (Fig. 5H). Nevertheless, using more specific markers, in 15% of hemisegments (*n*=52) an ectopic Eg/En-coexpressing NB was observed at the position of NB6-4 in mutant MN (Fig. 5N,N′). Other NBs were never restored in gnathal segments of *Df(3L)H99* mutants. These data suggest that, with the exception of mandibular NB6-4, PCD does not significantly account for the reduction in NB numbers in gnathal segments.

### Deformed suppresses the formation of specific NBs in gnathal segments

Because the homeotic gene *Deformed* (*Dfd*) is expressed in MN and MX (Fig. 5I,J) (McGinnes et al., 1998), we tested its role in shaping the gnathal NB pattern. In *Dfd^16^* mutant embryos, we found a slightly increased number of Dpn^+^ NBs in MX (Fig. 5H). Using more specific NB markers (e.g. Eg, Ey, En, Ind, Lbe, Vnd, Wg) we often detected an ectopic (Wg^+^) NB at the position of NB5-4 (81% of hemisegments, *n*=26; Fig. 5E-G′) and, in one case (*n*=48 hemisegments), an ectopic Eg^+^ NB at the position of NB2-4 (Fig. 5C) in the mutant MX. Furthermore, in the mutant MN, an ectopic Eg/En-coexpressing NB at the position of NB6-4 was found in 20% of hemisegments (*n*=40; Fig. 5A,B). However, NBs were never restored in the anterior mandibular compartment, where, in the wild type, the reduction of the NB pattern is most substantial. Thus, in this region Dfd does not seem to play a role in suppressing NB formation, in accordance with our observation that during the period of NB formation Dfd is already largely downregulated in the neuroectoderm of the anterior mandibular compartment (Fig. 5I). In rare cases, an ectopic Eg^+^ NB at the position of NB2-4 was also detected in LB of *Dfd^16^* mutants (2/48 hemisegments; Fig. 5D). Since in wild type we detected Dfd protein in the neuroectoderm from which labial NB2-4 seems to develop (Fig. 5I, inset), this suggests that Dfd acts cell-autonomously to suppress labial NB2-4 formation.

As we observed an ectopic mandibular NB6-4 both in *Df(3L)H99* and *Dfd^16^* mutants, we tested in *Dfd^16^* mutants whether this is due to a decrease in PCD in the mandibular neuroectoderm. We observed a substantially reduced number of Dcp-1^+^ cells in MN and MX neuroectoderm (Fig. 5K-M), including the En-expressing dorsal neuroectoderm from which the ectopic mandibular NB6-4 develops: in wild type, Dcp-1 was detected in 31% of hemisegments (*n*=64), versus 8% of hemisegments (*n*=26) in *Dfd^16^* mutants (Fig. 5K, inset). Thus, Dfd positively regulates PCD in neuroectodermal progenitor cells and, thereby, suppresses formation of the mandibular NB6-4. By contrast, the maxillary NB5-4, which was often restored in *Dfd^16^* mutants (see above), was never found in *Df(3L)H99* mutants (*n*=24 hemisegments). These findings suggest that Dfd does not exclusively act via PCD to restrict NB formation.

### The number of neuroectodermal progenitors differs between gnathal segments

Since PCD does not significantly contribute to segment-specific restriction of NB numbers in the gnathal segments, we next examined whether it is related to the number of neuroectodermal progenitors from which those NBs develop. Upon staining against Msh (Drop – FlyBase) and En, neuroectodermal cells were counted in all four spatial quadrants of each gnathal and prothoracic hemineuromere at early stage 9, when expression of En (in posterior cells) and Msh (in dorsal cells) is sufficiently established, and before onset of PCD (Fig. 5O). Compared with T1, the total cell number was reduced in MX (by ∼15%) and MN (by ∼43%), but not in LB. Whereas in MX cell numbers were diminished only in the anterior quadrants, in MN a reduction was found in all four quadrants, but most significantly in the anterodorsal quadrant (by ∼64%; Fig. 5P). These findings match the NB pattern of both segments, where NBs preferentially in anterodorsal positions do not form. Therefore, early determination of smaller segmental sizes of gnathal neuroectodermal anlagen appears to mainly define the numbers of delaminating NBs. The various regions within segmental neuroectodermal anlagen (e.g. the anterior compartment of MN) are differentially affected by size reduction.

### Modifications in DV gene expression establish an expanded domain of proneural gene expression to allow for rapid formation of NBs in the MN

**Fig. 6.**
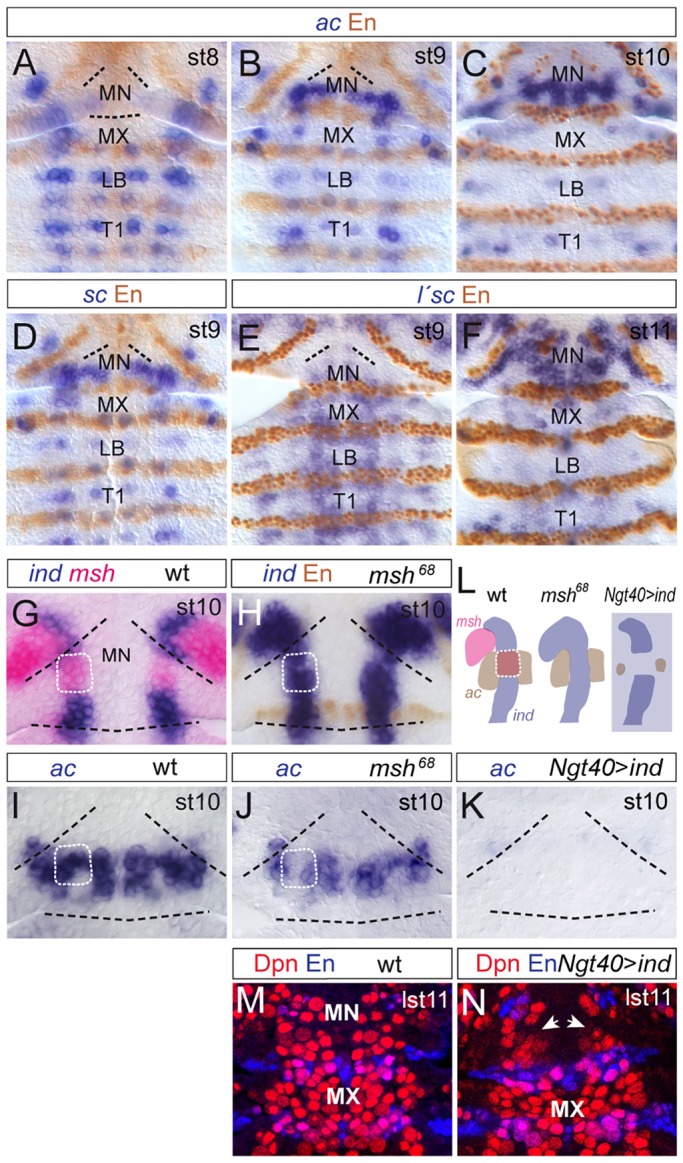
**Proneural gene expression in gnathal segments and its segment-specific modification by the activity of DV genes (*ind*, *msh*).** (A) Stage 8, showing delayed onset of *ac* expression in MN. (B,C) During stage 9/10 *ac* is expressed in a large domain in MN. (D) Expression of *sc* is similar to that of *ac*. (E,F) At stage 11 *l'sc* expression is downregulated in MX and LB, but is prominent in anterior MN. Black lines (A,B,D,E) indicate the segmental border of MN. (G-L) Stage 10. Black dashed lines indicate segmental borders and white dotted frames indicate corresponding regions of intermediate neuroectoderm. Ectopic *ind* expression in intermediate mandibular neuroectoderm in *msh^68^* (H) or *Ngt40>ind* (K) embryos represses *ac* (J,K). (L) Summary of G-K (see main text for details). (M,N) Composite confocal images of Dpn and En labeling in wild-type (M) and *Ngt40>ind* (N) embryos at late stage 11. Upon *ind* overexpression many row 3, 4, 5 NBs and MP2s (arrows), which would normally develop from the mandibular *ac* expression domain (see Fig. S2.6A′,B′), are lacking.

Although NB formation is completed by late stage 11, we find that many NBs of the MN (∼60%), particularly in the anterior compartment, are delayed compared with the other gnathal segments (Fig. 1). Accordingly, most mandibular NBs develop in a fairly narrow time window and, as shown above, from a comparatively small neuroectoderm. To see if this peculiarity is reflected in the activity of proneural genes we investigated the expression of *ac*, *sc* and *lethal of scute* (*l'sc*; *l(1)sc* – FlyBase) in the neuroectoderm. The early spatiotemporal expression of these genes in LB, MX and the posterior compartment of MN is similar to the situation in truncal segments, where each NB develops from a small proneural cluster of neuroectodermal cells (Fig. 6F-K) (Jiménez and Campos-Ortega, 1990). In the anterior compartment of MN, however, proneural gene expression starts slightly later, in accordance with the postponed development of many anterior NBs (Fig. 1). Strikingly, during stages 9 to 11, *ac* and *sc* are continuously expressed in this compartment at high levels in an oversized, neuroectodermal stripe (Fig. 6F-I), from which (by stages 10/11) eight to nine NBs develop in a narrow time window; these include part of row 3, 4, 5 NBs and the MP2-like NBs (Fig. 6A-C). Expression of *l'sc* begins slightly later than that of *ac*/*sc* and, until stage 11, when it is already downregulated in MX and LB, it is expressed within a large domain covering the MN (Fig. 6J,K). Unlike *ac* and *sc*, *l'sc* is also expressed in the most anterior neuroectoderm of MN, suggesting that development of the most anterior NBs (i.e. row 2) depends on L'sc activity.

In contrast to posterior segments, in which *ac* and *sc* are expressed in dorsal and ventral neuroectoderm (Skeath et al., 1994), in the anterior MN expression of both genes also covers intermediate neuroectoderm, suggesting a segment-specific regulation by DV genes. It has been shown that Ind is a direct repressor of *ac* in truncal segments (Zhao et al., 2007a) and that Msh is a repressor of *ind* expression (Zhao et al., 2007b; Seibert et al., 2009). We found that, specifically in the anterior MN, *msh* is also expressed early in the intermediate neuroectoderm where it keeps *ind* suppressed and, thereby, enables expression of *ac* (Fig. 6L,N). Accordingly, in *msh^68^* mutants *ind* is derepressed (83% of hemisegments, *n*=36) and *ac* is repressed in the anterior-intermediate mandibular neuroectoderm (70% of hemisegments, *n*=28) (Fig. 6M,O). Additionally, upon overexpression of *ind* (using the maternal driver *Ngt40-*Gal4), *ac* expression was significantly reduced in the neuroectoderm (82% of hemisegments, *n*=40; Fig. 6P,Q), often followed by a variable loss of Dpn^+^ NBs in rows 3, 4, 5 and the MP2s (50% of hemisegments, *n*=40; Fig. 6R,S). Thus, segmental modifications in DV gene expression establish an expanded domain of *ac* expression to enable the rapid and consecutive formation of many NBs from the comparatively small neuroectoderm in the anterior MN.

## DISCUSSION

### NB maps of the gnathal segments complete the characterization of segmental patterns and individual identities for all NBs building the CNS of the fly

**Fig. 7.**
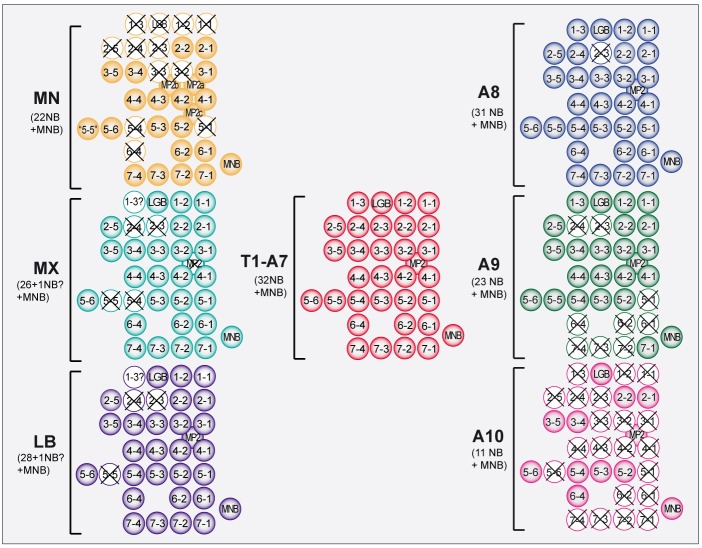
**Comparison of the NB pattern in gnathal, thoracic and abdominal segments.** The scheme illustrates NBs that are formed (colored) or missing (X) in the left hemineuromere of mandibular (MN), maxillary (MX) or labial (LB) segment and terminal abdominal segments (A8, A9, A10; according to Birkholz et al., 2013a), as compared with the ground state pattern (according to Doe, 1992), which is repeated in all segments from T1 to A7.

Whereas substantial knowledge has accumulated regarding the characteristics of neural stem cells and the generation of cell diversity in the truncal (thoracic/abdominal) CNS, relatively little is known about the developing gnathal neuromeres, which represent a peculiar transitional tissue in that they form the most anterior part of the larval VNC which becomes associated with the adult brain. In this study we describe the spatiotemporal pattern of NB formation in the gnathal segments (stages 8 to 11) and provide the first comprehensive maps of 46 marker genes expressed in subsets of NBs by late stage 11. At that stage the segmental units of head and trunk are most clearly displayed, and the final pattern of NBs is established. With the NB maps presented here for the gnathal region, characterization of the entire population of neural stem cells building the VNC and brain (except the optic lobes) of the fly is completed (in total 2×567 NBs), each of these cells now being individually identified (Doe, 1992; Urbach et al., 2003; Birkholz et al., 2013a; this study, see Figs 4 and 7); this number, however, does not include the mesectodermal midline progenitors (Bossing and Technau, 1994; Bossing and Brand, 2006; Wheeler et al., 2006), which remain to be determined in the gnathal and pregnathal segments (except MNB, this study).

Remarkably, almost all identified gnathal NBs represent serial homologs of NBs in the thoracic/abdominal segments, and some of them of NBs in the brain (as discussed below), which is reflected by the neuroectodermal position and time point of their formation, by the NB-specific code of identity genes (see Table S2) and, as shown for some of the gnathal NBs, by the production of typical cell lineage components. As shown in previous studies, some serially homologous thoracic/abdominal NBs produce modified segment-specific lineages due to differential specification of NBs and their progeny, differences in proliferation and/or PCD (for reviews, see Rogulja-Ortmann and Technau, 2008; Technau et al., 2006). For the NB4-2 lineage, we show that, departing from the situation in the thorax, the RP2 motoneuron undergoes PCD in the late embryonic LB and MX, whereas it seems not to be formed in MN. Concomitantly, the NB4-2-specific marker gene code reveals differences in each gnathal segment in terms of *Dfd*, *Sex combs reduced* (*Scr*) and *cap-n-collar* (*cnc*) expression; further, *CenG1A* is tagma-specifically expressed in gnathal NB4-2, and *cas* and *chrb* only in mandibular NB4-2. These divergently expressed factors may represent candidates that confer the segment-specific characteristics to their lineages. Modifications in the expression profile are found among many serially homologous gnathal NBs, but particularly in mandibular NBs. Therefore, we expect lineage divergences to be most pronounced in MN.

The completed NB map sets the stage for systematic cell lineage analysis. By comparison with the known lineages of the thoracic and anterior abdominal neuromeres, such an analysis will uncover the degree of conservation of serially homologous lineages in derived segments. It will also allow an investigation of the impact of differences in the molecular signature of their stem cells on the presence or absence of particular progeny cells.

### Regulation of the segment-specific patterns of gnathal NBs

Hox genes play a crucial role in segmental identity and patterning, in part by regulating PCD (reviewed by Rogulja-Ortmann and Technau, 2008; Reichert and Bello, 2010). In gnathal segments, PCD has been reported to occur at segmental boundaries (Nassif et al., 1998). It is required for the maintenance of a normal boundary between maxillary and mandibular lobes and is induced by Dfd, which activates the pro-apoptosis gene *reaper* (Lohmann et al., 2002). Here, we have shown that Dfd suppresses the formation of particular gnathal NBs in different ways: in case of mandibular NB6-4 by inducing localized PCD in neuroectodermal progenitors that constitute the NB6-4 proneural cluster; in case of maxillary NB5-4 in a PCD-independent manner, possibly by repressing NB-promoting genes (i.e. proneural genes). So far we do not know if Scr plays a role in suppressing NB formation in the labial segment. Recently, the Hox genes *Abdominal B* and *caudal* have been reported to suppress the development of specific NBs in the terminal abdominal segment in a PCD-independent manner (Birkholz et al., 2013b).

Only a few NBs are restored in *Dfd^16^* mutants (NB6-4 in MN, NBs 2-4, 5-4 in MX, NB2-4 in LB). These do not include NBs in the strongly reduced anterior compartment of MN. Since Dfd protein is downregulated until stage 11 specifically in the neuroectoderm of this compartment (Fig. 5I) (see also McGinnis et al., 1998), it might not affect the formation of the comparatively late-developing anterior NBs. As PCD also plays only a minor role, it is likely that NB numbers are mainly determined by the smaller size of gnathal segmental anlagen (for blastodermal fate map see Hartenstein et al., 1985), which include lower numbers of neuroectodermal progenitors, especially in the anterior MN, where the decrease in NBs is most apparent. Different sizes of the gnathal neuroectodermal anlagen are assumed to be defined by early patterning genes acting along the DV (e.g. *dpp*, *sog*, *dorsal*) and AP (e.g. gap genes *hb*, *btd*, *gt*) axes (Cohen and Jürgens, 1990; Reinitz and Levine, 1990; Stathopoulos et al., 2002; Mizutani et al., 2005). Finally, we cannot exclude the possibility that homeotic genes (i.e. *Dfd*, *Scr*) are also involved, although the size of the early gnathal neuroectoderm seems unaltered in *Dfd* mutants.

Despite the substantial reduction in the number of neuroectodermal progenitor cells in the anterior MN, many NBs develop within a short time window from an oversized proneural domain that constantly expresses high levels of *ac* and *sc*. Considering its extent and the relatively high number (eight to nine) NBs emerging from this proneural domain, we assume that these NBs originate from adjacent neuroectodermal progenitor cells, similar to the situation in the central brain (Urbach et al., 2003; Kunz et al., 2012). This contrasts with the situation in MX, LB and truncal segments, where proneural clusters are small and where only one cell per cluster adopts an NB fate. Thus, spatial and temporal patterns of proneural gene expression, and presumably the modes of NB formation, differ between gnathal segments. We show that the oversized *ac* domain in MN is established by a segment-specific alteration in DV gene expression. It is likely that other factors are also involved in the regionalization and maintenance of *ac* and *sc* expression. For example, the cephalic gap gene *btd* (Younossi-Hartenstein et al., 1997), *gt* (expressed similarly to *ac* in anterior MN; Fig. S2.1D), *cnc* or *col* (both specifically expressed in MN; McGinnis et al., 1998; Crozatier et al., 1999) are potential regulators.

### Segmental deviation from the NB ground state pattern – comparison of gnathal and terminal abdominal segments

The pattern of embryonic NBs in the thoracic (T1-T3) and anterior abdominal (A1-A7) segments resembles the ground state (T2) (Lewis, 1978). Segmental divergence from this ground pattern is obvious in all gnathal (this study) and terminal abdominal (A8-A10) (Birkholz et al., 2013a) segments, as summarized in Fig. 7. In the gnathal segments the population of NBs is progressively diminished in the anterior direction, i.e. from LB to MN (∼3, ∼5 and 12 NBs are missing in LB, MX and MN, respectively). In the abdominal tail region it is progressively diminished in the posterior direction, i.e. from A8 to A10 (one, nine and 21 NBs are missing in A8, A9 and A10, respectively). Notably, in the terminal abdominal segments (A9, A10) NBs are lacking preferentially in the posterior compartment (Birkholz et al., 2013a), whereas in the gnathal region NBs are missing preferentially in the anterior compartment of each segment. Nevertheless, some NBs appear to be preferred victims of segmental modification in gnathal as well as in terminal abdominal segments: NB2-3 is missing in all gnathal segments and in A8-A10, and NB2-4 is missing in all gnathal segments and A9, A10. Conversely, seven NBs can be consistently identified in all of these segments (NBs 2-1, 2-2, 3-4, 3-5, 5-2, 5-3, 5-6), and thus are more resistant to segmental modifications in NB patterns. Nevertheless, several of these ‘conserved’ NBs generate lineages that have been shown to differ among gnathal, thoracic and abdominal segments with regard to the number and/or particular types of progeny cells, and there are indications that these differences primarily affect the later parts of their lineages (Bossing et al., 1996; Schmidt et al., 1997; Schmid et al., 1999; Baumgardt et al., 2009; Karlsson et al., 2010). Thus, the early parts of their lineages might form repetitive units of the neuronal network, the specific functions of which might be required in all truncal and gnathal neuromeres.

### Linking postembryonic lineages to embryonic NBs in the gnathal CNS

After a period of mitotic quiescence, a specific subset of embryonic NBs resumes proliferation during larval development to produce adult-specific neurons (reviewed by Maurange and Gould, 2005). In thoracic and abdominal segments, embryonic NBs are reactivated in a segment-specific pattern (Truman and Bate, 1988), which is already determined by Hox genes in the embryonic neuroectoderm (Prokop et al., 1998). In the gnathal segments, a substantial number (>80%) of embryonic NBs disappears during late embryonic and larval development, most of them due to PCD. Only ∼14 NBs and their corresponding lineages (13 paired, 1 unpaired) have been detected in the gnathal region of the late larva, most of which develop in LB (Kuert et al., 2014). Each of the eight to nine postembryonic lineages in LB could already be linked to a specific embryonic NB, which demonstrated that in LB and abdominal segment A1 (with a likewise reduced set of reactivated NBs) they emerge from a similar set of serially homologous embryonic NBs (Birkholz et al., 2015). Serial homologs of most of these NBs also exist in embryonic MX and MN (e.g. NBs 4-2, 5-2, 6-1, 6-2, 7-1, 7-4, MNB), which are likely to include the precursors of the remaining (approximately six) postembryonic lineages described by Kuert et al. (2014) for the gnathal region. As four of those lineages express En (Kuert et al., 2014), they presumably stem from embryonic En-expressing NBs in rows 6 and 7 (NBs 6-1, 6-2, 7-1, 7-4).

The postembryonic lineages in the VNC of the larva can be individually identified based on their morphological characteristics (Truman et al., 2004, 2010) and specific codes of gene expression (e.g. Lacin et al., 2014; Li et al., 2014), and all of them have been linked to identified embryonic NBs (Birkholz et al., 2015). Notably, sets of transcription factors found to be expressed in specific postembryonic lineages (Lacin et al., 2014) often differ from those expressed in their parent embryonic NBs, revealing highly dynamic expression of these factors.

### Serially homologous NBs in neuromeres of the abdomen/thorax, gnathal and pregnathal head

In a previous report we gave a first example for a serially homologous NB in neuromeres of trunk (abdomen/thorax) and brain (Urbach et al., 2003). Here we elaborated on these investigations by comparing the characteristics of all NBs in neuromeres of the thorax, gnathal and pregnathal head, the latter giving rise to the brain (trito-, deuto- and protocerebrum). Remarkably, almost all NBs in the tritocerebrum seem to represent serial homologs of NBs in the gnathal and thoracic/abdominal segments. Furthermore, the population of tritocerebral NBs is likely to represent a subset of NBs found in MN and, in turn, those in MN, MX and LB represent a subset of those in the next posterior segment (MX, LB and T1), respectively. This suggests that intersegmental modification of the NB pattern in the three gnathal and the tritocerebral neuromeres involves a progressive reduction compared with the thoracic ground state. Also, approximately half of the deutocerebral NBs display potential serial homology to NBs in the gnathal and thoracic neuromeres, but not all of them have a counterpart in the tritocerebrum. Notably, serial homology is not overt in the population of (∼70) protocerebral NBs that constitute the anterior and largest part of the brain. This is in agreement with recent data showing that expression patterns of postembryonic lineages in the VNC are not consistently linked to the expression of lineages in the central brain (Li et al., 2014). Together, the appearance of serially homologous NBs seems to mirror the order in which segmental patterning of the neuroectoderm, and the formation and specification of NBs, is genetically conserved: conformity to the thoracic ground state is highest in LB and progressively diverges from MX to the protocerebrum (this study; Crozatier et al., 1999; Seibert et al., 2009; reviewed by Urbach and Technau, 2004, 2008). The existence of NBs in the brain showing serial homology to those in the VNC is astounding considering that NB formation is significantly delayed in MN and tritocerebrum (Fig. 1) (Urbach et al., 2003), and that segmental patterning of the neuroectoderm as well as the formation of NBs is regulated by different mechanisms in the brain (Hirth et al., 1995; Younossi-Hartenstein et al., 1997; Seibert et al., 2009; Urbach and Technau, 2004).

These intersegmental comparisons not only uncover serial homologies among NBs based on similarities, but also segmental modifications in their expression profiles. Since nearly all of the marker genes considered in this study encode transcription factors, these modifications are likely to be responsible for the establishment of segment-specific characteristics of serially homologous NB lineages. Thus, the NB map forms a basis for comprehensive analyses of the lineages building the CNS of the fly and presents candidate factors controlling their region-specific composition. Some of these factors are presumably part of the genetic network that provides instructions for the establishment of neural circuits involved in the processing of feeding and taste response. This work might therefore also help to decipher the genetic mechanisms that direct gnathal-specific behavior.

For the first time the entire complement of NBs in a complex animal has been mapped. Combined with the detailed gene expression studies in this and previous reports this provides a framework for understanding the mechanisms underlying segmental patterning of the CNS. It will also serve as a reference and provide valuable tools for comparative analyses of neurogenesis in other species.

## MATERIALS AND METHODS

### Fly strains

The fly strains used in this study are listed in the supplementary Materials and Methods.

### Immunohistochemistry and *in situ* hybridization

Embryos were dechorionated, fixed, immunostained and flat preparations performed according to published protocols (Patel, 1994; Jussen and Urbach, 2014). The antibodies used are detailed in the supplementary Materials and Methods. Probes for *in situ* hybridization were synthesized using the templates shown in Table S4. *In situ* hybridization was performed as described previously (Jussen and Urbach, 2014) and probes processed with NBT/BCIP solution for non-fluorescent staining (Carl Roth) or tyramide signal amplification (TSA Cyanine 3 System; PerkinElmer) for fluorescent stainings. Embryos were then immunolabeled with primary antibody followed by incubation with biotinylated (processed with DAB) or fluorescent dye-coupled secondary antibodies as described in the supplementary Materials and Methods. Non-fluorescent stainings were documented on a Zeiss Axioplan microscope, while fluorescent confocal images were acquired on a Leica TCS SP5 II microscope. Images were processed with ImageJ (NIH), Adobe Photoshop and Adobe Illustrator.
